# Hæmorrhoids: Causes and Symptoms

**Published:** 1896-01-25

**Authors:** A. G. Miller

**Affiliations:** Lecturer on Clinical Surgery, Senior Surgeon Edinburgh Royal Infirmary.


### Medical Progress and Hospital Clinics.

[*The Editor will be glad to receive offers of co-operation and contributions from members of the profession. All letters should be addressed to* The Editor, at the Office, 428, Strand, London, W.C.]

### HÆMORRHOIDS—CAUSES AND SYMPTOMS.

By A. G. Miller, M.D., F.R.C.S.E., Lecturer on Clinical Surgery, Senior Surgeon Edinburgh Royal Infirmary.

In order to understand properly the causes, symptoms, and treatment of piles (or hæmorrhoids), we must have some idea of what they are. They may be briefly and simply defined as dilated and congested veins near the anus (inferior hæmorrhoidal plexus), which cause a protusion of the mucous membrane or skin covering them, and are accordingly designated internal and external piles respectively. They might equally correctly be termed submucous and subcutaneous, the line of demarcation between them being the junction of the mucous membrane of the rectum with the skin of the perineum at the anus. The subcutaneous external pile contains simply a dilated and congested vein (varicose), which may become inflamed and thrombosed, the superimposed skin, however, does not become implicated, unless the pile suppurates—a result which is very rare in my experience. The submucous internal pile, however, is sometimes a more complex structure, having cavernous tissue as well as veins in its composition. Its mucous membrane is closely adherent, and cannot be separated from it. Moreover, it is liable to suppuration and ulceration, with consequent tendency to bleeding. Submucous piles, that occur just at the margin of the anus, are simply venous, like the subcutaneous ones, while those higher up generally partake of the more complex structure. The former are dark purple, while the latter are dark red (raspberry) in colour.

Piles may occur singly, or they may be multiple. Not unfrequently one sees what seems to be a complete circle of them surrounding or protruding from the anus.

Some persons have loose folds of skin at the anus, which are often called external piles. They are not, correctly speaking, piles at all; they are only the remains of external piles, which have inflamed and become obliterated by thrombosis of the dilated veins, which once distended the skin over them. With these short introductory remarks let me take up the subject of the causes of piles.

*A.* Piles may be caused by anything that gives rise to obstruction to the venous return from the rectum and anus. The causes may be divided into (1) predisposing, and (2) exciting. A more practical division would be into those producing congestion and dilatation of the veins, and those irritating the already-formed piles. What used to be called "a fit of the piles" was an attack of inflammation in piles that most probably pre-existed. (1) The predisposing causes, then, or those things that tend to produce congestion of the inferior hæmorrhoidal veins, are simply conditions that obstruct the venous return. Foremost among these is constipation. Indeed, I have always found that patients suffering from piles are constipated. However it acts I shall not attempt to explain, but certainly lodgment of fæces in the large intestine seems to cause dilatation of the veins of the lower part of the rectum and anus. I shall not occupy your time either by detailing the anatomical peculiarities which have been described as predisposing causes. These you will find in your text-books. (2) Exciting causes, or irritants, are easily found. There is the passage of the hard rough fæces produced by the constipation. Then there may be straining at stool, and the use of paper after stool with some irritating material on it, such as ink. Lastly, there may be irritation from septic causes. One constantly finds that persons suffering from piles are habitually constipated (congestion), and get relief only once or twice a week (a very common thing in women) by medicine, when they are freely purged (irritation); and they will tell you that they do not suffer from their piles except after the bowels have acted. This combination and alternation of constipation and purging is, in my opinion, the most frequent cause of piles, as also (as we shall see) of fissure of the anus. Again, septic infection may cause inflammation in previously congested hæmorrhoidal veins. The absorption of sepsis may occur directly through the skin or mucous membrane, or may be acquired indirectly through the circulation. Two questions occur at this stage—Are piles hereditary? Are they constitutional? I cannot enter into this subject fully. I shall merely state that, in my experience, the tendency to piles and varicose veins seems to "run in families," and what causes piles in some persons does not produce them in everybody—as, for instance, pregnancy. Piles may have other causes when they are merely a symptom, as in cancer of the rectum and cirrhosis of the liver. Of these conditions I will speak again when I come to the subject of diagnosis.

*B.* We may now consider the symptoms of piles. The first is itching at the anus. This draws attention to the part, when a slight enlargement or protrusion may be felt. This itching means irritation, dilatation having been present previously, as we have already seen. If the irritation be not removed inflammation follows, and then we have another symptom, namely, pain. The lump which, when previously felt, was soft is now hard, because the vein has become thrombosed. In this condition the pile is intensely painful, especially if external. In this latter case the patient cannot walk or sit properly. He has no rest except when lying in bed on his side. But even then there is a burning aggravating pain that is very trying and wearing out, and a motion of the bowels is looked forward to with horror. If the pile be left to itself it will continue to cause pain for some days, and then symptoms will gradually subside and there will remain a memory of suffering, or, perhaps, if the pile be an external one, a tag of skin at the anus. What happens is that the thrombus in the inflamed vein becomes absorbed; very rarely, in my experience, does it break down and give rise to an abscess.

